# Subcutaneous extraskeletal osteosarcoma of foot: A case report

**DOI:** 10.1016/j.ijscr.2020.09.062

**Published:** 2020-09-22

**Authors:** Keykhosro Mardanpour, Mahtab Rahbar

**Affiliations:** a Kermanshah Medical Science University, Iran; b Iran Medical Science University, Hemmat Boulevard, Postal code: 1765463, Tehran, Iran

**Keywords:** Osteosarcoma, Subcutaneous, Plantar, Foot

## Abstract

**Introduction:**

Subcutaneous extraskeletal osteosarcoma (ESO) is a rare high grade malignant mesenchymal neoplasm consistent of approximately 1% of soft tissue sarcoma. Less than 10% of ESOs are superficial, originating in subcutis.

**Presentation of case:**

We reported a 72 years old male with chief complain of a painless slowly growing soft tissue mass in his plantar aspect of right foot in the last 9 months before admission. The plain radiography showed a large subcutaneous soft tissue mass in plantar area and heel, Magnetic resonance images displayed an irregular subcutaneous soft tissue mass extending from posterior lower leg to ankle and involving calcaneus inferiorly. A percutaneous core biopsy of the mass, obtained. The histopathology examination confirmed an soft tissue osteosarcoma. Subsequent wide local excision of the mass performed. Final histopathological result was subcutaneous extraskeletal osteosarcoma.

**Discussion:**

The patient has had no evidence of recurrence or/and distant metastasis at 25 months followed up. However, excising subcutaneous ESOs with a safe surgical margin is paramount for achieving the best outcome.

**Conclusion:**

A subcutaneous ESO is a rare malignant tumor. We recommended that along with clinicohistological findings, radiological correlation is necessary for ruling out other diagnoses.

## Highlights

• Subcutaneous extraskeletal osteosarcoma is a rare high grade mesenchymal sarcoma.
• The exact diagnosis can be established with histologic study.
• Wide marginal resection is associated with a decreased risk of local recurrence and metastasis.

## 1 Introduction

Extraskeletal osteosarcomas (ESOs) accounts for 1%–2% of all soft tissue sarcomas and approximately 2%–4% of all osteosarcomas [1]. It typically affects patients between 50 and 70 years of age. The reported male to female ratio is 1.9:1 [2]. ESO arises in the soft tissue, may involve the periosteum, cortex, or medullary canal secondarily. The pathogenesis of ESOs is unknown. The majority develops de novo. However, about 10% of ESOs may be radiation or trauma-induced. The prognosis of ESOs is poor [3]. The most subcutaneous extraskeletal osteosarcomas (SESOs) are deep-seated in the dermis or subcutis. The most common location of ESOs in the lower extremity (75%; thigh and buttock), followed by the upper extremity (15%–23%, shoulder girdle), and the retroperitoneum (17%). The most common clinical presentation of ESOs is a progressively enlarging soft tissue mass, rarely causing pain or tenderness [4]. We report a 76-year-old farmer man with a SESO embedded within the subcutaneous soft tissue and fat pad of the right foot. Also, we discuss the relevant clinicopathologic presentation, diagnosis, and treatment Informed consent obtained from the patient before the preparation of this manuscript. Our study has been reported in line with the SCARE criteria [5].

## 2 Presentation of case

**Fig. 1:**
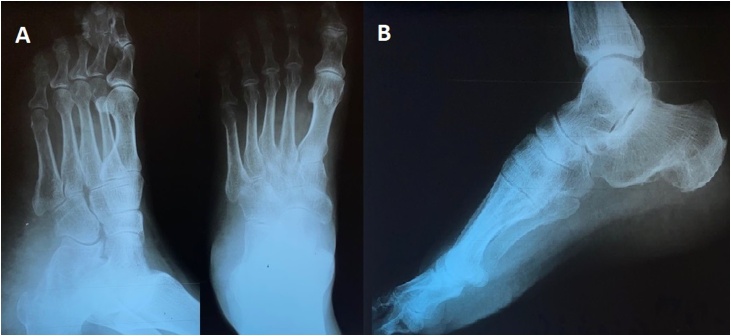
Plain radiograph: Anteroposterior views show soft tissue mass in plantar and heel of right foot (A), lateral view shows subcutaneous soft tissue mass in foot plantar and heel with slight calcaneus periosteal irregularity of calcaneus bone (B).

**Fig. 2:**
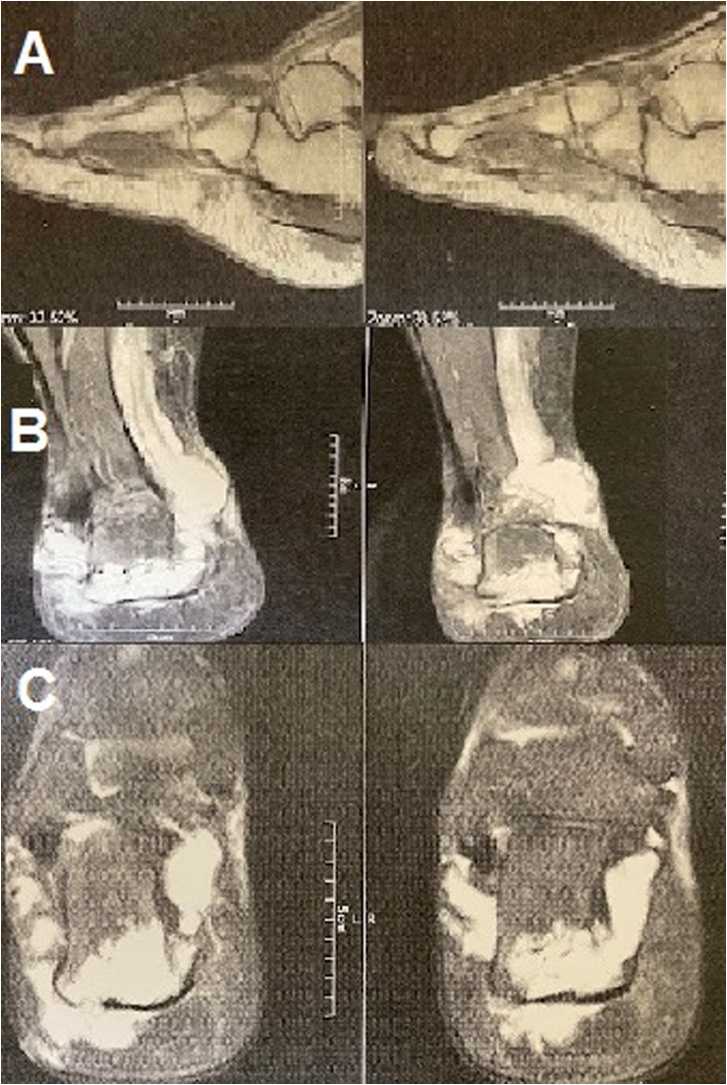
T1- and T2-weighted MRI in sagittal (A, B) and axial (c) planes demonstrating a large soft tissue mass superficial to the deep plantar fascial plane and embedded within the subcutaneous and deep dermis tissue. The lesion has a low signal intensity on T1weighted images (A) and heterogeneous signal intensity on T2-weighted images (B, C).

**Fig. 3:**
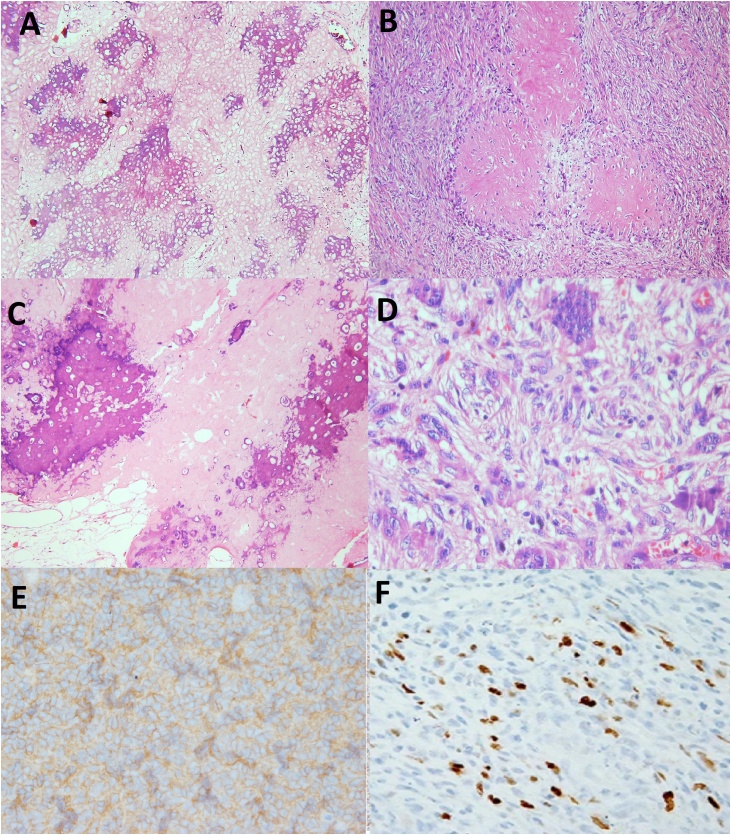
Microscopic appearance of subcutaneous soft tissue tumor shows osteoid formation surrounded by malignant osteoblastic cells without interposition of cartilage (A, B) (HE, ×100), (C) (HE, ×200), neoplastic cells with marked atypia (pleomorphic, hyperchromatic) and bizarre giant tumor cells noted (D) (HE, ×200). Immunohistochemistry staining: cytoplasmic positive immunoreactivity of CD99 in tumoral cells of osteosarcoma (IHC ×200) (E), cytoplasmic positive immunoreactivity of osteocalcin in tumoral cells of osteosarcoma (IHC ×200) (F).

**Fig. 4:**
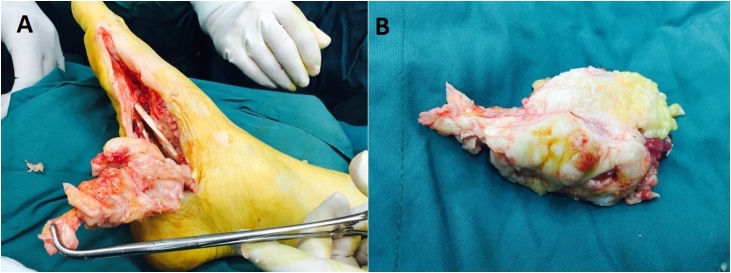
Intraoperative macroscopic appearance of subcutaneous soft tissue tumor indicates tumor was marginally resected and preserved the major neuro-vascular bundles, tendons and muscles (A, B).

A 72-year-old healthy farmer man presented with a painless slowly growing mass in his plantar aspect of right foot in May 2018. At first, the small soft tissue mass grew in the foot plantar location for approximately 6 months ago and then slowly developed in his heel during nine months ago. He is a farmer in the north of Iran, sometimes he worked on his farm with hard shoes or even without shoes in paddy rice. He told a persistent history of previous trauma to his foot plantar for a long time. Additionally, no history of cancer elicited. On physical examination, an 11 × 4.5 × 3 cm firm and irregular mass palpated on his right foot plantar that extended to heel overlying with unremarkable skin. The plain radiography of foot showed a large subcutaneous soft tissue mass in plantar area and heel, possibly with peripheral chunky calcification (Fig. 1). Magnetic resonance images (MRI) displayed an irregular subcutis soft tissue mass extending from posterior lower leg to ankle, hind foot, and forefoot laterally and involving calcaneus inferiorly. The images showed heterogeneous and relatively hypointense signal intensity on T1-weighted images and hyperintense signal intensity on T2 (Fig. 2). The lesion did not invade subjacent bone or muscle but extended into subcutaneous areas and providing extensive subcutis edema. Computed tomography (CT) of the chest, abdomen, and pelvis did not show metastatic or other lesions. Routine laboratory testing was unremarkable. The clinical and imaging findings were suggestive of a malignant soft tissue mass. The patient referred to our institute for biopsy. Due to the large size of the lesion, a core biopsy performed. Histopathology examination showed a malignant neoplasm characterized by the proliferation of anaplastic spindle cell with the presence of lacy osteoid matrix and immature bone. The mitotic activity (>10 mitoses per 10 high-power fields), and atypical mitotic figures noted. Immunohistochemistry analyses indicated that the tumor cells expressed CD99 (85%), osteocalcin (90%), and vimentin (95%) and didn't express smooth muscle actin (SMA), desmin, S100 protein, EMA, pan-cytokeratin, and PLAP. The stain for the Ki-67 analogous MIB-1 shows high proliferative activity with values around 30% (Fig. 3). The patient was diagnosed with subcutaneous extraskeletal osteosarcoma as stage II, grade 3 (according to the World Health Organization system). The patient received adjuvant chemotherapy and radiotherapy preoperatively, although ESOs are relatively chemoresistant compared to osseous osteosarcomas. Four weeks later, the wide localized resection of the mass performed included an 8 cm skin graft. This resection included excision of the deep plantar fascia below the lesion. Grossly, the tumors were hemorrhagic and focally necrosis and firmly attached to the fascia, and it lied in contact with the periosteum of the calcaneus bone. The connective tissue capsule surrounded the tumor and adhered to the subjacent structures that made surgical dissection difficult (Fig. 4). However, the tumor infiltrates to the subcutis. There was no evidence of overlying skin ulceration. Histologically, all the mass sections showed 95% tumor necrosis. (Percentage of tumor necrosis, as follows: a good responder, ≥90%, or a poor responder, <90%). Two weeks after surgery, a postoperative MRI showed only edema which was related to soft tissue congestion. No areas of suspicious enhancement or inguinal lymphadenopathy were seen.

The patient did not receive postoperative chemotherapy or radiation. Six months later, a repeat MRI was performed which showed no resolution of the postoperative changes and no evidence of recurrent or residual tumor. The patient has had no evidence of recurrence or/and distant metastasis at 25 months followed up as verified with single-photon emission computed tomography/positron emission tomography (SPECT CT/PET).

## 3 Discussion

Extraskeletal osteosarcomas most frequently occur in patients older than 40 years old. All the subtypes of osteosarcoma of bone have reported in ESOs. Osteoplastic ESO is the most common variant followed by fibroblastic ESO. Well-differentiated ESO is a rare variant [6]. Several studies documented that the immunophenotype of ESO is similar to osteosarcoma of bone. The CD99 marker associated with alkaline phosphatase expressed in all types of osteosarcoma, but osteocalcin is the most specific antigen for ESOs that expressed in the malignant cells and matrix in 82% of cases. ESOs are uniformly positive for vimentin, 68% express smooth muscle actin, 25% desmin, 20% S100 protein, 52% EMA, 8% keratin, and 0% PLAP. The stain for the Ki-67 analogous MIB-1 shows high proliferative activity with values around 25%. Systematic genetic differences between extraskeletal and bone osteosarcomas have not documented [7,8] The previous analysis highlighted several factors associated with a better outcome include size less than 5 cm (the most important), fibroblastic or chondroblastic histological subtype, and diminished proliferative activity as measured by Ki-67 index. However, the validity of these prognostic factors is controversial [9,10]. The prognosis of ESO patients is poor, and the 5-year survival ranges from 10% to 46%. More than 50% of the patients experience multiple local recurrences and distal metastases. Distal metastases are usually to the lungs (>80%), followed by the regional lymph nodes, bone, brain, liver, and skin. Response to treatments (mainly chemotherapy) is worse [11]. Our case underwent a wide margin excision. The patient was free of recurrence or metastasis for about 25 months since the primary resection. Although the size of the tumor was large, chemoradiotherapy and wide marginal excision of tumor thought to contribute to his survival length.

## 4 Conclusion

A subcutaneous ESO is a rare malignant mesenchymal tumor of the soft tissue. We recommended that along with clinicohistological findings, radiological correlation is necessary for ruling out other diagnoses. However, pre-operative chemoradiotherapy associated with wide excision of subcutaneous ESOs is paramount for achieving the best outcome.

## Declaration of Competing Interest

The authors report no declarations of interest.

## Funding

No source of funding.

## Ethical approval

Ethical approval was not required in the treatment of the patient in this report.

## Consent

Written informed consent was obtained from the patient forpublication of this case report and accompanying images. A copy of written consent is available for review by Editor-in-Chief of this journal on request.

## Author contribution

Keykhosro Maedanpour: writing the manuscript, study design, final review.

Mahtab Rahbar: data collector, writing the manuscript.

## Registration of research studies

N/A.

## Guarantor

Mahtab Rahbar is the guarantor and accepts full responsibility.

## Provenance and peer review

Not commissioned, externally peer-reviewed.

## References

[1] Curfman K.R., Morrissey S.L. (2019). Extraskeletal osteosarcoma recognized following acute traumatic injury. Case Rep. Oncol..

[2] Mirabello L., Troisi R.J., Savage S.A. (2009). Osteosarcoma incidence and survival rates from 1973 to 2004: data from the Surveillance, Epidemiology, and End Results Program. Cancer.

[3] Saadaat R., Abdul-Ghafar J., Ud Din N., Haidary A.M. (2020). Anal extraskeletal osteosarcoma in a man: a case report and review of the literature. J. Med. Case Rep..

[4] Mardanpour K., Rahbar M. (2008). Calcaneal Osteosarcoma; a Case Report. Iran. J. Med. Sci..

[5] Agha R.A., Borrelli M.R., Farwana R., Koshy K., Fowler A., Orgill D.P., For the SCARE Group (2018). The SCARE 2018 statement: updating consensus surgical CAse REport (SCARE) guidelines. Int. J. Surg..

[6] Hoch M., Ali S., Agrawal S., Wang C., Khurana J.S. (2013). Extraskeletal osteosarcoma: a case report and review of the literature. J. Radiol. Case Rep..

[7] Salomão D.R., Schmitt N.J., Wenger D.E., Schlafmann S., Fritchie K. (2019). Extraskeletal osteosarcoma: a rare case arising in phthisis bulbi with a review of the literature. Ocul. Oncol. Pathol..

[8] Tan K.T., Idowu O.K., Chandrasekar C.R., Yin Q., Helliwell T.R. (2012). Extraskeletal osteosarcoma of the hand. Hand (N Y).

[9] Xin S., Wei G. (2020). Prognostic factors in osteosarcoma: a study level meta-analysis and systematic review of current practice. J. Bone Oncol..

[10] Bispo Júnior R.Z., Camargo O.P. (2009). Prognostic factors in the survival of patients diagnosed with primary non-metastatic osteosarcoma with a poor response to neoadjuvant chemotherapy. Clinics (Sao Paulo).

[11] Narayanan S.T., Gopalakrishnan M.K., Ibrahim S.S., Sankar R. (2016). Extraskeletal osteosarcoma - a case report. J. Clin. Diagn. Res..

